# Aprepitant versus ondansetron in preoperative triple-therapy treatment of nausea and vomiting in neurosurgery patients: study protocol for a randomized controlled trial

**DOI:** 10.1186/1745-6215-13-130

**Published:** 2012-08-03

**Authors:** Sergio Bergese, Adolfo Viloria, Alberto Uribe, Alejandra Antor, Soledad Fernandez

**Affiliations:** 1 Ohio State University Medical Center, N411 Doan Hall, 410W 10th Ave, Columbus, OH, 43210, USA; 2 Ohio State University Medical Center, 2012 Kenny Road, Columbus, OH, 43210, USA

**Keywords:** Aprepitant, Craniotomy, Ondansetron, Postoperative nausea and vomiting

## Abstract

**Background:**

The incidence of postoperative nausea and vomiting (PONV) is 50% to 80% after neurosurgery. The common prophylactic treatment for postoperative nausea and vomiting is a triple therapy of droperidol, promethazine and dexamethasone. Newer, more effectives methods of prophylaxis are being investigated. We designed this prospective, double-blind, single-center study to compare the efficacy of ondansetron, a neurokinin-1 antagonist, and aprepitant, as a substitute for droperidol, in the prophylactic treatment of postoperative nausea and vomiting after neurosurgery.

**Methods:**

After obtaining institutional review board approval; 176 patients, 18 to 85 years of age with American Society of Anesthesiologists (ASA) classifications I to III, who did not receive antiemetics 24 h before surgery and were expected to undergo general anesthesia for neurosurgery lasting longer than 2 h were included in this study. After meeting the inclusion and exclusion criteria and providing written informed consent, patients were randomly assigned in a 1:1 ratio to one of two treatment groups: aprepitant or ondansetron. The objective of this study was to conduct a randomized, double-blind, double-dummy, parallel-group and single-center trial to compare and evaluate the efficacies of aprepitant versus ondansetron. Patients received oral aprepitant 40 mg OR oral dummy pill within 2 h prior to induction. At induction, a combination of intravenous dexamethasone 10 mg, promethazine 25 mg, and ondansetron 4 mg OR dummy injection was administered. Therefore, all patients received one dummy treatment and three active PONV prophylactic medications: dexamethasone 10 mg, promethazine 25 mg, and either aprepitant 40 mg OR ondansetron 4 mg infusion. The primary outcome measures were the episodes and severity of nausea and vomiting; administration of rescue antiemetic; and opioid consumption for 120 h postoperatively. Standard safety assessments included adverse event reports, physical and laboratory data, awakening time and duration of recovery from anesthesia.

**Discussion:**

The results of this comparative study could potentially identify an improved treatment regimen that may decrease the incidence and severity of postoperative nausea and vomiting in patients undergoing neurosurgery. Also, this will serve to enhance patient recovery and overall satisfaction of neurosurgical patients in the immediate postoperative period.

**Trial registration:**

Registered at The Ohio State University Biomedical Sciences Institutional Review Board: Protocol Number: 2007 H0053

## Background

The overall incidence of postoperative nausea and vomiting (PONV) is around 30% and as great as 70% to 80% in high-risk individuals [1]. For patients undergoing neurosurgery the PONV incidence is about 50% to 80%. Indicators for increased risk of PONV with craniotomy surgery includes: infratentorial lesion, female gender and children older than 2 years of age [2]. Although PONV is rarely fatal, it does increase morbidity by threatening wound dehiscence, hematoma formation, aspiration, esophageal rupture, dehydration, and increases in intraocular and intracranial pressures due to acute blood pressure elevations [1]. In addition, patients in the early postoperative period after craniotomy surgery experiencing hypertension have an increased mortality if they develop intracranial hemorrhage [3]. Multiple willingness to pay studies have shown that patients desire most to avoid pain and nausea or vomiting, and this has over-ridden even the fear of a catastrophic anesthetic outcome [4,5]. Research shows patients are willing to pay $100 out of pocket to prevent nausea and vomiting [6]. Therefore, PONV results in patient discomfort and dissatisfaction, the need for a larger nurse-to-patient ratio, increased post-anesthesia care unit (PACU) times, unintended admissions, extended postoperative stays and increased overall healthcare costs [1].

PONV risk can be predicted from three characteristics: the patient, the procedure and the pharmacotherapy/anesthesia technique. The patient qualities that increase PONV risk are: female gender (after puberty), younger age (which tapers after puberty), non-smoking status, obesity, anxiety, and a history of PONV/motion sickness [1]. Length of surgery increases the incidence by approximately 60% from baseline for each 30 minutes of operative time [1]. Surgical procedures that increase risk include: plastic surgery/breast augmentation, middle ear, tonsillectomy, strabismus, laparoscopic, gynecological, and neurosurgery (particularly infratentorial craniotomies) [1,2]. Anesthesia techniques can augment PONV outcomes. General anesthesia increases risk of PONV 11-fold compared with regional anesthesia [1]. Likewise, less PONV is associated with awake craniotomy compared to general anesthesia [7]. Induction and maintenance of anesthesia with propofol and/or avoiding inhalational anesthetics reduces PONV. Presurgical hydration with 15 to 20 ml/kg of crystalloid can reduce the incidence of PONV. Neuromuscular reversal with neostigmine, especially in doses greater than 2.5 mg can cause PONV. Avoiding opioids and using alternative modalities for analgesia (that is, non-steroidal anti-inflammatory drugs (NSAIDs) such as acetaminophen and ibuprofen) can reduce the risk of PONV. Oxygen supplementation during the intraoperative period can cut the risk of PONV by half [1]. A PONV risk factor chart was developed by Apfel *et al*. in 1999, identifying four main risks: female gender, non-smoker, history of PONV, and the use of perioperative opioids [8]. The presence of zero, one, two, three, or four of these risk factors equates to 10%, 20%, 40%, 60%, and 80% risk of PONV, respectively [8]. The general consensus is to prophylactically treat those patients with moderate to high risk for PONV [9].

Despite these findings, PONV is still a common complication of anesthesia and the number one cause of unanticipated admission after surgery [1,10]. Emesis pathophysiology is multifactorial, incited by drugs, environment, radiation and disease states [11,12]. The act of vomiting is controlled by multiple areas within the central nervous system (CNS), including the medulla, vestibular apparatus, and cerebral cortex [12]. The medulla contains the area postrema (AP), the dorsal motor nucleus of the vagus, and the nucleus tractussolitarius (NTS), collectively referred to as the ‘emesis center’. Additionally, the vestibular apparatus feeds auditory stimuli and positional changes into the vomiting center, while cortical structures allow emotions, tastes, sights and smells to play a role [11]. Numerous signals must arrive at the emesis center in an appropriate sequence to stimulate emesis [12]. When these stimuli converge, outputs activate the lower esophageal sphincter (LES) and stomach, sympathetic ganglia, respiration, swallowing, and baroreceptors. The resulting sensation of nausea is thought to originate in the cerebral cortex. Vomiting is a complex process that empties the gastrointestinal tract by retrograde phasic contractions [12]. At least 17 neurotransmitters have been detected in the AP and NTS.

Specific receptors including opioid, dopamine, histamine, cannabinoid, serotonin, acetylcholine, and neurokinin 1 (NK-1) are associated with the vomiting reflex [12]. Therapeutic options have been aimed at blocking or augmenting many of these receptors. The first class of antiemetics is dopamine antagonists of which metoclopramide and droperidol are examples. Metoclopramide (Reglan), a prokinetic agent that increases muscle tone of the lower esophagus sphincter and enhance gastric emptying, is likely to cause nervous system side effects such as jitteriness, insomnia, sedation, or anxiety with large doses. Droperidol, a very effective antiemetic, received a US Food and Drug Administration (FDA) ‘black box’ warning due to potential life threatening arrhythmias and clinically relevant prolongation of the QTc interval at high doses [1,13]. Antihistamines (that is, promethazine, cycline, hydroxyzine, and diphenhydramine) block histamine receptors in the vomiting center, but frequently cause sedation and dry mouth. Anticholinergic scopolamine patches have the ability to prevent emesis for up to 72 h, but frequently cause sedation, blurred vision, dry mouth and confusion [6,14]. Serotonin antagonists (that is, ondansetron, dolasetron, and granisetron) are most effective when given towards the end of surgery, though rare side effects include headache, constipation and increased liver enzymes [1,14]. The anti-inflammatory agent dexamethasone a synthetic adrenocortical steroid, used prophylactically before the induction of anesthesia appears to be an effective antiemetic. Side effects similar to chronic steroid use, adrenal insufficiency and immunosuppression, have not been associated with a single dose [1,15].

For those patients at moderate to high risk, combination therapy has shown to be more efficacious for PONV prophylaxis. A common triple therapy regimen includes droperidol, promethazine, and dexamethasone, which allows smaller doses to be as effective when dosed together [1]. Triple therapy practice was affected by the increased warnings and contraindications for droperidol issued by the FDA. Droperidol is not currently recommended or used in standard combination therapy for PONV. Furthermore, PONV can occur up to 5 days after surgery, and most antiemetic therapy would need frequent redosing [14].

A future approach to preventing PONV is blockade of substance P and NK-1 receptors. Substance P, named for the powdered product discovered by Gaddum and Schild, has been studied since the early 1900s [16]. Current research has established substance P as a prominent neurotransmitter released from both the CNS and the peripheral nervous system (PNS) afferent neurons. Substance P interacts with NK receptors, which are rhodopsin-like structures coupled to G-proteins. This interaction of neurotransmitter and receptor is involved in many disease processes including: asthma, chronic bronchitis, inflammatory bowel disease, cystitis, migraines, seizures, pain, depression, and emesis [16]. Substance P activity occurs at primary sensory neurons in the PNS, and produces an inflammatory reaction referred to as neurogenic inflammation.

More recent research is targeting the relationship between substance P and the CNS. Animal studies demonstrated that application of substance P to the emesis center induced vomiting. Substance P is likely involved in decreasing gastric pressures, slowing antral motility and relaxing the LES, all involved in the act of regurgitation [12]. Few human trials exist examining NK-1 antagonists in the prevention of PONV, showing a decreased incidence. Of further interest, the NK-1 antagonist aprepitant may have antiemetic effects as far out from surgery as 48 h, important with post-discharge nausea and vomiting and opioid induced emesis [14,17]. The FDA-approved aprepitant, an oral NK-1 antagonist, for treatment and prevention of cisplatin-induced nausea and vomiting.

## Methods

We conducted a randomized, double-blind, double-dummy, parallel-group, single-center study of high risk for PONV patients to investigate the administration of triple antiemetic prophylactic therapy. The objective of this study was to compare and evaluate the efficacies of aprepitant versus ondansetron for prophylactic combination therapy in preventing nausea and vomiting in neurosurgery patients. The safety of the two triple therapies in these patients was also assessed.

The standard PONV prophylaxis regimen for high-risk patients at Ohio State University Medical Center (OSUMC) consists of dexamethasone, promethazine, and ondansetron. In this study, we used a double-dummy design to substitute aprepitant for ondansetron in a 1:1 ratio to determine the relative efficacies of these two drugs in combination therapy. The study groups were compared for the number of patients that develop PONV, the number and total dose of rescue from PONV medications given postoperatively, and patient satisfaction with their treatment. We hypothesized a significantly greater percentage of neurosurgery patients will experience no vomiting during the immediate 48-h postoperative period in the prophylactic aprepitant triple therapy group.

Adult patients, 18 to 85 years of age, scheduled for neurosurgery requiring opening of the cranium and dura at OSUMC and who consent in writing to participate in this study were eligible. Patients who were (1) prisoners, (2) pregnant women, (3) mentally ill, (4) under the age of 18 or over the age of 85, (5) graded as American Society of Anesthesiologists (ASA) classification V (6) alcohol or drug abusers or (7) had a cerebral perfusion pressure (CPP) greater than 150 mmHg or less than 50 mmHg were excluded from this study.

After receiving approval from The Ohio State University institutional review board, 176 consecutive patients who meet the inclusion and exclusion criteria and who give written informed consent to participate in the study were randomly assigned by the research pharmacist to 1 of 2 experimental groups using a 1:1 ratio. All study personnel were blinded to group assignment. Patients in group I received 25 mg promethazine intravenously, 10 mg dexamethasone intravenously, 4 mg ondansetron intravenously, and an inactive oral dummy pill. Patients in group II received 25 mg promethazine intravenously, 10 mg dexamethasone intravenously, 40 mg aprepitant orally, and an inactive dummy intravenous injection. Thus, all patients received 25 mg promethazine and 10 mg dexamethasone. Because this is a double-blind, double-dummy study and ondansetron was given intravenously whereas aprepitant was given orally, it was necessary to give patients an oral or intravenous placebo, depending on their group assignment, for uniformity and to maintain the double-blindedness. This study does not contain a placebo arm, though dummy treatment using placebo was necessary due to the different routes of medications. Therefore, each patient received the three drugs in the PONV prophylactic triple cocktail, plus an intravenous or oral dummy treatment prior to induction of anesthesia (Table 1). Funding for these medications was provided internally, through the Department of Anesthesiology.and preoperative data about each patient in the two groups were recorded (Table 2). The duration of each surgery (anesthesia time) will be recorded for each patient. Patients were continuously monitored in the PACU, surgical intensive care unit (SICU) and the medical floor for a total of 120 h postoperatively. Episodes of nausea, vomiting and administration of rescue therapy for either nausea or vomiting were recorded and time stamped by blinded personnel. In addition, the severity of the nausea or vomiting was recorded. Nausea will be reported by the patient and evaluated by the blinded personnel utilizing a standard verbal response scale (VRS) ranging from 0 to 10, 0 being no nausea and 10 being severe nausea. Vomiting will be evaluated numerically by the blinded personnel as either 0, no vomiting, 1, mild vomiting, 2, moderate vomiting, or 3, severe vomiting. Rescue therapy for PONV episodes consisted of 4 mg ondansetron. After the first 24 h of starting the triple therapy antiemetic, an electrocardiogram (ECG) was recorded as well as blood drawn for analysis.

**Table 1:** Postoperative nausea and vomiting (PONV) medications and administration

**Group I**	**Group II**
Preoperative PONV medications:	
4 mg ondansetron intravenously + placebo PO	40 mg aprepitant PO + intravenous placebo
25 mg Promethazine intravenous	25 mg Promethazine intravenous
10 mg dexamethasone intravenous	10 mg dexamethasone intravenous
Postoperative PONV rescue therapy:	
Multiple 4 mg ondansetron	Multiple 4 mg ondansetron

**Table 2:** Demographic and preoperative data

**Demographic data**	**Preoperative data**
Gender	Systolic, diastolic and median blood pressure
Age	Electrocardiography (ECG) recording
History of postoperative nausea and vomiting (PONV)	Anesthesia modality
Surgery	Renal function
Smoking history	Hepatic function
Race	Cerebral perfusion pressure (CPP)
Motion sickness history	
Past reactions to the study drugs	

### Efficacy variables

Efficacy variables were collected based on previously designed PONV studies [18,19]. The primary efficacy variable is the percentage of patients with no vomiting over 0 to 72 h postoperatively across the two treatment groups.

The secondary efficacy variables are as follows: (1) proportion of patients with a complete response during delayed (24 to 120 h; days 2 to 5) and overall (0 to 120 h; days 1 to 5) after neurological surgery and general anesthesia; (2) proportion of patients with complete control, defined as no emetic episode, no need for rescue medication and no more than mild nausea overall (0 to 120 h; days 1 to 5) after neurological surgery and general anesthesia; (3) assessment of the severity of nausea and vomiting during acute (0 to 24 h), delayed (24 to 120 h) and overall (0 to 120 h) intervals after neurological surgery and general anesthesia; (4) assessment of the time to treatment failure (defined as time to first emetic episode and/or to first use of rescue medication); (5) assessment of the time to first emetic episode; (6) assessment the time to significant nausea (defined as nausea rated ≥4 on a 0 to 10 verbal response scale or nausea that required rescue therapy).

The incidence of any adverse reaction to treatment in our two experimental groups was recorded. In the ondansetron-treated patients (group I), all cardiovascular, gastrointestinal, hepatic, integumentary and neurologic postoperative adverse events was recorded and analyzed for cause. For instance, treatment-related diarrhea, headaches, fever, akathisia and acute dystonic reactions was also recorded and analyzed. Similarly, in the aprepitant patients (group II) all adverse events related to the digestive, hemic, lymphatic, nervous, cardiovascular and respiratory systems was recorded and analyzed for cause.

### Statistical methods

From Gan *et al*. [18] who compared the use of aprepitant 40 mg to ondansetron 4 mg in a similar PONV study involving patients undergoing open abdominal surgeries and an overnight hospitalization, 85% of the patients experienced no vomiting to aprepitant treatment and 67% to ondansetron treatment. For a two-sided test to compare the two proportions at 0.05 significance level, we need n = 88 per group to achieve 80% power. We sought n = 100 subjects per group to account for screening and attrition in the study.

The efficacy data was analyzed based on the intention-to-treat principal. Demographic and other patient characteristics for all randomized patients were reported by descriptive statistics, such as: mean, standard deviation, and so on. Also, we examined the comparability of the two treatment groups with respect to important preoperative factors. Logistic regression was used to test the primary hypothesis with demographic characteristics as potential covariates in the model. For the number of rescue therapy treatments used during the 120-h postoperative period, Wilcoxon rank sum test was performed. For other secondary endpoints, the analysis plan was similar to the one for the primary endpoint. Standard safety assessments included adverse event reports, physical and laboratory data, awakening time and duration of recovery from anesthesia.

## Discussion

This study could potentially identify an improved prophylaxis for PONV for patients undergoing neurosurgery. This treatment will decrease the incidence and severity of PONV and enhance the overall comfort and satisfaction of neurosurgical patients in the immediate postoperative period. We anticipate that this study would have a moderate to high rate of screen failures not only due to changes or lack of adherence to the triple therapy, but due to changes in patients’ condition, physician preferences, or intolerance to the medications. Another issue is referred to the lack of adequate assessments for postoperative nausea and vomiting due to patients lost to follow-up.

## Trial status

100 patients were enrolled for this study. Of the 100 patients, were 24 screening failures. Data collection for the 76 eligible patients was completed and recorded. Since this a double blind study, statistics and data analysis on this study need to be completed.

## Competing interests

The authors declare that they have no competing interests to report.

## Authors’ contributions

SB, the principal investigator, designed the current study, drafted the manuscript, oversaw revisions, and gave final approval on all aspects of the study. AV, AU, AA, and SF were involved with the acquisition of data, drafting and revision of the manuscript, analysis and interpretation of available results. All authors read and approved the final manuscript

## Authors’ information

SB received a MD degree from the National University of Cordoba, Argentina in 1988. In 1991, he completed an internship in the Department of Surgery at the Italian Hospital in Cordoba, Argentina. From 1992 to 1997, SB worked as a Research Associate in the Department of Surgery, Division of Transplantation at The Ohio State University where he completed a fellowship in Cellular Immunology and Vascular Biology. He proceeded to do a residency in general surgery, and later completed his residency in the Department of Anesthesia at The Ohio State University. In 2008, he obtained a Certificate of Business Administration. Presently, he is Associate Professor, Director of Neuroanesthesia and the Neuroanesthesia Fellowship, and Director of Clinical and Neurological Research in the Department of Anesthesia at The Ohio State University. SB’s main research interests and expertise are neuroanesthesia, anesthesia monitoring, and mitigation of risks and discomforts associated with anesthesia. As director of a relatively large clinical trials research group, SB has mentored numerous research and neuroanesthesia fellows and currently oversees more than 50 projects in the areas of anesthesia, neurosurgery, general surgery, and critical care. In 2006, he was selected as America’s Top Anesthesiologist. He is an active member of the ASA, SNACC, IARS, SAMBA, and ASRA. SB also serves on the editorial board of several journals including the *Journal of Neurosurgical Anesthesiology*, *World Journal of Anesthesiology* and *International Journal of Medicine*. He has been a faculty at more than 50 national and international meetings, where he has presented over 200 lectures. SB also has over 40 publications and over 150 abstracts presented at national and international conferences.

## References

[B1] Gan TJ Meyer T Apfel CC Chung F Davis PJ Eubanks S Kovac A Philip BK Sessler DI Temo J Tramer MR Watcha M Watcha M Consensus guidelines for managing postoperative nausea and vomiting Anesth Analg 2003 97 62 71 12818945 10.1213/01.ane.0000068580.00245.95

[B2] Audibert G Vial V Postoperative nausea and vomiting after neurosurgery (infratentorial and suprtentorial surgery) Ann Fr Anesth Reanim 2004 23 422 427 10.1016/j.annfar.2004.01.005 15120791

[B3] Basali A Mascha EJ Kalfas I Schubert A Relationship between perioperative hypertension and intracranial hemorrhage after craniotomy Anesthesiology 2000 93 48 54 10.1097/00000542-200007000-00012 10861145

[B4] Macario A Fleisher LA Is there value in obtaining a patient’s willingness to pay for a particular anesthetic intervention? Anesthesiology 2006 104 906 909 10.1097/00000542-200605000-00003 16645439

[B5] Macario A Weinger M Carney S Kim A Which clinical anesthesia outcomes are important to avoid? The perspective of patients Anesth Analg 1999 89 652 658 10475299 10.1097/00000539-199909000-00022

[B6] Gan TJ Postoperative nausea and vomiting - can it be eliminated? JAMA 2002 287 1233 1236 10.1001/jama.287.10.1233 11886298

[B7] Manninen PH Tan TK Postoperative nausea and vomiting after craniotomy for tumor surgery: a comparison between awake craniotomy and general anesthesia J Clin Anesth 2002 14 279 283 10.1016/S0952-8180(02)00354-9 12088812

[B8] Apfel CC Kranke P Eberhart LHJ Roos A Roewer N Comparison of predictive models for postoperative nausea and vomiting Br J Anaesth 2002 88 234 240 10.1093/bja/88.2.234 11883387

[B9] Murphy MJ Hooper VD Sullivan E Clifford T Apfel CC Identification of risk factors for postoperative nausea and vomiting in the perianesthesia adult patient J Perianesth Nurs 2006 21 377 384 10.1016/j.jopan.2006.09.002 17169747

[B10] Fortier J Chung F Su J Unanticipated admission after ambulatory surgery: a prospective study Canadian J Anaesth 1998 45 612 619 10.1007/BF03012088 9717590

[B11] Watcha MF White PF Postoperative nausea and vomiting. Its etiology, treatment, and prevention Anesth 1992 77 162 184 10.1097/00000542-199207000-00023 1609990

[B12] Prommer E Aprepitant (EMEND) The role of substance P in nausea and vomiting J Pain Palliat Care Pharmacother 2005 19 31 39 16219609

[B13] Charbit B Albaladejo P Funck-Brentano C Legrand M Samain E Marty J Prolongation of QTc interval after postoperative nausea and vomiting treatment by droperidol or ondansetron Anesthesiology 2005 102 1094 1100 10.1097/00000542-200506000-00006 15915019

[B14] Kovac AL Prevention and treatment of postoperative nausea and vomiting Drugs 2000 59 213 243 10.2165/00003495-200059020-00005 10730546

[B15] Henzi I Walder B Tramer MR Dexamethasone for the prevention of postoperative nausea and vomiting: a quantitative systemic review Anesth Analg 2000 90 186 194 10.1097/00000539-200001000-00038 10625002

[B16] Harrison S Geppetti P Substance P Int J Biochem Cell Biol 2000 33 555 576 11378438 10.1016/s1357-2725(01)00031-0

[B17] Gan TJ Kovac A Lubarsky DA Phillip BK PONV Management: Tackling the Practical Issues. CME/CE 2005–2006 http://www.medscape.com/viewprogram/4990

[B18] Apfel CC Roewer N Korttila K How to study postoperative nausea and vomiting Acta Anaesthesiol Scand 2002 46 921 928 10.1034/j.1399-6576.2002.460801.x 12190791

[B19] Gan TJ Apfel C Kovac A Phillip B Lawson F The NK1 receptor antagonist aprepitant for prevention of postoperative nausea and vomiting Anesthesiology 2005 103 A769

